# Electroacupuncture with extensor exercise improves the contraction elastic density of quadriceps in short and long term for knee osteoarthritis

**DOI:** 10.1007/s10067-024-07243-5

**Published:** 2024-11-25

**Authors:** Bingfeng Xing, Yuanyuan Liu, Xin Zhou, Guanheng He, Wenya Pei, Zhanmou Liang, Jingwen Ruan, Yinghua Duan

**Affiliations:** 1 The First Affiliated Hospital/The First Clinical Medicine School of Guangdong, Pharmaceutical University, Guangzhou, China; 2 The First Affiliated Hospital, Sun Yat-Sen University, Guangzhou, China

**Keywords:** Elastic shear wave imaging, Electroacupuncture (EA), Extensor training, Knee osteoarthritis (OA), Quadriceps femoris, WOMAC scoring scale

## Abstract

**Background:**

Extensor training improves the quadriceps contraction intensity of knee osteoarthritis. But the duration of effects is limited. This study aimed to assess whether electroacupuncture (EA) with extensor training (EA + E) has better short-and long-term effects than simple extensor training (E).

**Methods:**

Fifty-four patients were categorized into EA + E and E groups. Elastic shear wave imaging was employed to ascertain the quadriceps elastic modulus values (EMV) at different time points and extension angles. WOMAC scale was used to measure knee function.

**Results:**

Compared to before EA, the vastus lateralis (VL) EMV with 180° knee extension immediately after EA increased significantly. After 1 month, the vastus medialis (VM) EMV with 180° extension increased and WOMAC scores decreased significantly than immediately after EA. The EMV of the VM and VL of group EA + E increased, and WOMAC scores decreased significantly compared to group E after 1 month. After 6 months, EMV of the VM and VL in EA + E group significantly increased, and WOMAC scores significantly decreased compared to E group.

**Conclusions:**

In short- and long-term, EA with extensor training enhances the contraction strength of the quadriceps and knee function for OA.

**Trial registration:**

This prospective single-center randomized controlled trial was approved by the Review Committee of the First Affiliated Hospital of Sun Yat-sen University (Registration date: 28/02/2023, Ethical Number: [2023] 005; The Clinical trial registration number: ChiCTR2300076651.

**Key Points:**

• *The contraction strength of the quadriceps femoris muscle in OA was detected with shear wave elastic ultrasound, and electroacupuncture immediately enhanced the contraction strength.*

• *After the completion of electroacupuncture treatment, the quadriceps strength and the function of knee joint in OA can be continuously improved, which will last for 6 months.*

• *In short and long term, electroacupuncture with extensor training has better enhancement of quadriceps contraction strength of and knee function for OA than extention only.*

## Introduction

Osteoarthritis of the knee (OA), also known as degenerative arthritis, is a chronic degenerative osteoarthritis characterized by the degeneration of articular cartilage, inflammation of surrounding soft tissues, secondary joint hyperostosis, and joint deformity. The primary pathogenesis of the disease involves the progression of arthritis, leading to the destruction of the articular cartilage matrix, reduction in the number of chondrocytes and cartilage debris, and induction of inflammation, fibrosis, and hyperostosis in surrounding soft tissues [1–3]. With China’s aging population, the incidence of OA has gradually increased. Its disability rate has reached 50% within 2 years of onset, seriously affecting patients’ quality of life and increasing the economic pressure on family and society for care and treatment [2, 3].

Currently, the main treatment approach for early to middle-stage OA is anti-inflammatory and pain relief methods, mainly using oral non-steroidal drug, which can cause severe damage to the gastrointestinal tract and are unsuitable for long-term use, with significant side effects [4]. However, drugs targeting cartilage repair, such as oral preparations containing Chondroitin and Glucosamine formula and intra-articular injection of viscous supplements, have also been controversial recently owing to insufficient clinical efficacy evidence [5, 6]. In the later stage of OA development, serious hyperostosis and even joint deformity often occur. The function of the knee joint is severely limited, requiring surgical treatment, such as arthroscopy and joint replacement of the knee, which greatly burdens the patients’ physiology, psychology, and family economy, and some patients will experience infection and relapse [7, 8]. In summary, effectively slowing down the progression of OA inflammation remains a complex clinical challenge.

Recently, the academic community has increasingly valued the significance of the morphology and function of the quadriceps in the occurrence and development of OA. Studies in the clinical and imaging domains of OA have found that the imaging manifestations of joints often lag behind the clinical symptoms, such as pain and fatigue [9]. The latest laboratory and clinical evidence indicates that muscle fibrosis and fat infiltration can lead to the decline of quadriceps contraction force. Conversely, the decline of quadriceps contraction force will aggravate the progress of OA inflammation [10, 11]. The decline of muscle contraction force correlates with the limitation of knee joint movement in the early-middle stage of OA. Particularly, the weakness of the quadriceps femoris can increase the incidence rate of OA imaging [12, 13]. In the case with severe imaging manifestations, decreased quadriceps contraction force exacerbates knee pain during walking [14]. Conversely, improving the contraction force of the quadriceps can significantly alleviate pain symptoms in OA [15]. In summary, exercise therapy to improve quadriceps contraction force has emerged as paramount conservative management for early middle-stage OA. However, its effect weakened significantly 2 months later [16], posing a huge challenge for the clinical treatment of this stage of OA. Maintaining quadriceps contraction force slows joint inflammation progress in the early middle stage of OA, and continuously improving the patients’ pain relief and joint function is significant for improving their quality of life. Therefore, it holds great clinical value to identify a treatment strategy that significantly improves quadriceps contraction force and knee joint range of motion in the early-middle stage and maintains this improvement through the treatment of OA.

Numerous studies have demonstrated that a 4-week course of electroacupuncture can significantly alleviate pain intensity associated with osteoarthritis (OA) and enhance joint function when compared to pharmacological interventions [17]. In terms of short-term analgesic effects, there is no substantial difference between electroacupuncture and percutaneous electroacupuncture [18]. These findings indicate that the immediate impact of electroacupuncture on OA is evident; however, given that OA represents a continuum of inflammatory progression, it remains an important scientific inquiry whether electroacupuncture can impede the advancement of OA and provide long-term benefits, as there are currently no systematic investigations addressing this issue. This study found that electroacupuncture (EA) combined with quadriceps training in patients with early to middle-stage OA improved quadriceps contraction force and knee function for at least 6 months. The specific research process is as follows:

## Methods

### Patients and exclusion criteria

According to the pre experiment, it is expected that the final effective rate of the treatment group will be 80%, while the control group will have a final effective rate of 30%. Unilateral test, *α*, is 0.05, and the ratio of sample size between the two groups is 1:1, *β* = 0.1. Verification efficiency is 1 − *β* = 0.9. *Z α* = 1.96, *Z β* = 0.84. The calculation result is *N* = 21 cases, with an expected dropout rate of 30%, so each group requires 21 + 21 * 0.3 ≈ 28 cases.

Sixty-two patients with degenerative knee arthropathy who met the inclusion criteria and were enrolled at the Acupuncture and Moxibustion Department of the First Affiliated Hospital of Sun Yat-sen University between March 2023 and June 2024 were included in the study. They were categorized into the EA + extensor training (E) and the E groups. The inclusion criteria were: (1) Meets the diagnostic criteria for degenerative joint disease: ① The enrolled patients comprised both male and female participants; ② Age ≥ 50 years old; ③ Morning stiffness < 30 min; ④ Knee examination indicates bone hypertrophy; ⑤ Bone sounds during joint activity; ⑥ Bone tenderness; ⑦ Radiological examination shows osteophyte formation; ⑧ No significant synovial membrane temperature increase. Patients with three of the aforementioned seven criteria were diagnosed with knee Osteoarthritis. (2) Patients who voluntarily participated in this randomized controlled trial signed an informed consent form. The exclusion criteria were as follows: (1) Central and peripheral nerve injuries and affected knee joints. (2) BMI index ≥ 28. (3) Primary muscular diseases include mitochondrial myopathy and periodic paralysis. (4) Severe renal or parathyroid dysfunction. (5) Patients with mental disorders cannot cooperate with treatment or examinations. (6) Pregnancy. (7) Other surgeries, such as arthroscopic or joint replacement, are performed during treatment or follow-up.

If severe shock, subcutaneous bleeding after acupuncture, and pain intolerance occur during the treatment period, the treatment program should be stopped and the study withdrawn.

### Randomization procedure

**Fig. 1:**
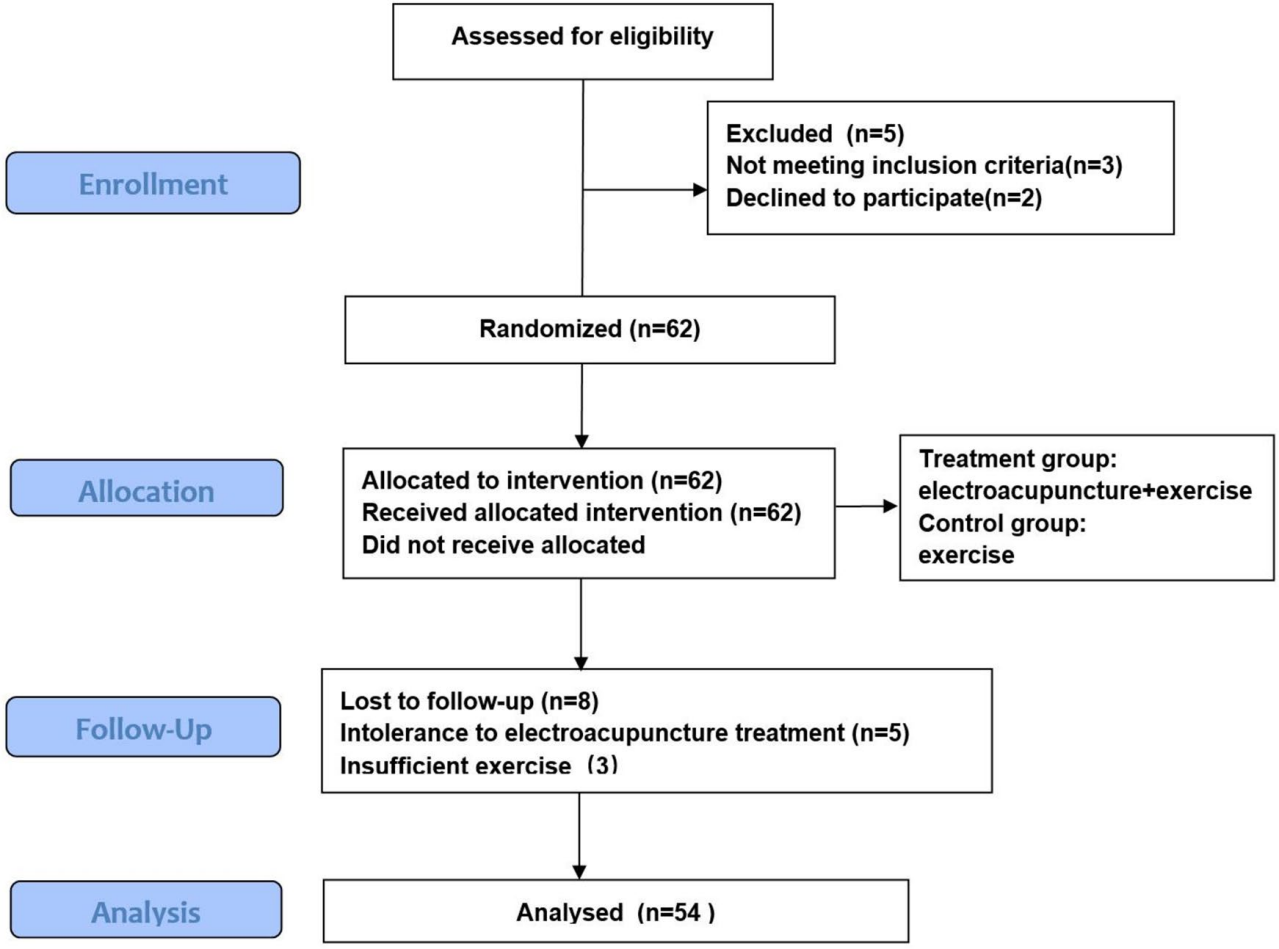
Transparent reporting of this trial

A computer-generated random number list was used to classify the patients into two groups randomly. Patients with odd random numbers were assigned to the EA + E group, while those with even random numbers were assigned to the E group. The random number allocation was performed by nurses who were not involved in the study. Blinding method target aimed to achieve the following: (1) Blinding of doctors providing exercise methods. Since both groups required exercise plans, all patients were guided by the doctors who provided the exercise methods after grouping. Accompanied by a nurse to ensure that group information is not leaked during the conversation to ensure that both groups receive the same exercise plan. (2) Blinding of B ultrasound doctors: Both groups underwent the quadriceps elasticity test, which was conducted with a nurse present to ensure that the grouping information was not disclosed in the conversation, and the B ultrasound doctor was blinded to patient grouping. At the conclusion of the study, eight patients withdrew from the study (five patients in the treatment group withdrew owing to EA intolerance, and three patients in the control group withdrew because of lack of compliance with the exercise protocol), with a final enrollment of 30 patients in the final treatment group and 24 in the control group. The CONSORT flowchart is as follows: (Fig. 1).

### Quadriceps exercise

Both groups performed quadriceps exercises (extensor training) for 30 min, thrice a week for 24 weeks. The specific methods are as follows: the patients were in a supine position, with the back of the lower limbs close to the bed surface. They tried to contract the quadriceps with extension of the knee joint and dorsiflexion of the ankle joint, held it for 5 s, relaxed for 5 s, and rotated it for 30 min. Group E underwent extensor training only. We followed the intervention requirements and regularly contacted the enrolled patients by phone, supervised quadriceps training, and required patients to provide exercise feedback during the next treatment.

### Quadriceps electroacupuncture

Electroacupuncture was applied to the following acupoints located within the projection of the muscle surface: XUEHAI (SP10), BAICHONGWO (EX-LE3)- VM, LIANGQIU(ST34), and YINSHI(ST33)-VL. The needle specification was 0.25 * 25 mm (Suixin brand, Guangzhou Suixin Medical Equipment Co., Ltd.), and the depth of each acupuncture point was 10 mm. Two acupuncture points on each muscle were connected to a pair of electrodes (Yingdi KWD-808-I electronic pulse acupuncture instrument; Changzhou Yingdi Electronic Medical Equipment Co., Ltd.). Electrical stimulation parameters: direct current, continuous wave, current intensity 0.8 ma, frequency 2 Hz. Treatment course: once a day, six times per course, for four courses; rest for 1 day between treatment sessions.

### Elastic modulus of quadriceps

The patients sat at the edge of the examination bed with their legs hanging naturally along the edge of the bed, not touching the ground. The ankles were separated by the same shoulder width, and the patient’s posture was adjusted until the tibial plateau was in the same plane as the bed edge. The elastic modulus of the VM and then the lateral thigh muscle was measured using a B-ultrasonic shear wave at knee flexion angles of 90°(FLE90), 45°(FLE45), and extended 180°(EXT180). During the examination, the relative position of the patient in the bed was kept fixed.

A coupling ultrasonic scanner (Mindaryresona7, Mindray Biomedical Electronics Co., Ltd, Shenzhen, China) with a linear transducer(5.6–10 Hz, Mindray Biomedical Electronics Co., Ltd, Shenzhen, China) was used in shear wave elastic mode to measure the Young’s modulus, assuming the isotropic nature of soft tissues. The shear modulus values were reported as Young’s modulus values because the skeletal muscle cannot be assumed to be isotropic. Each part was measured thrice by three experienced inspectors, and the elastic modulus value was averaged to reduce selection bias. The transducer was first oriented in the transverse plane to ensure that the right muscle was being measured. It was then rotated parallel to the fascicle direction. The optimal transducer location was determined when several muscle fascicles could be seen without disconnection through the image [19]. The standard surface of the muscle bundle long axis (uninterrupted) is VM or VL. A circle with a diameter of 10 mm was used as the sampling cross-section.

A virtual line was marked along the length of the thigh from the superior pole of the patella to the anterior superior iliac spine. The thigh circumference at 50% of the virtual line was measured. The VL was measured at 10% of the measured circumference distance laterally from the virtual line. Circumference was measured again at 20% distance from the virtual line. The VM was measured at 12.5% of the measured circumference distance, medially from the virtual line [20]. The shear modulus at each position was measured three times, and the average value was calculated.

The measurement points were marked with a surgical marker and checked every 5 days during treatment and follow-up. If any discoloration was observed, the nurse marked them again to avoid bias caused by measurement position deviation. The measurement time points were: before treatment, after 1 month, after 3 months, and after 6 months.

### Other bias control

Two groups of patients recorded their daily steps through a WeChat step-counting program. They did not engage in additional lower-limb weight-bearing exercises, such as brisk walking, running, and mountain climbing, to reduce the bias in the result caused by exercise. In addition, the clinical research data were entered into the computer by nurses, the E group and EA + E group are numbered as Group 1 and Group 2. Outcome assessors are blinded to the groups.

### Statistical analysis

All continuous data were expressed as mean ± standard deviation (upper and lower limits of 95% confidence interval) and analyzed using independent sample *t* tests. All discontinuous data expressed in frequency (percentage) were analyzed Pearson’s χ ^2^ or Fisher’s exact test. If the data did not conform to a normal distribution, the Moses test was used to check the distribution fit, and the *U*-test was used for analysis. The original significance levels was set to *α* = 0, and *P* < 0.05 indicated a significant difference.

## Result

### Baseline data

As shown in Table 1, the *X*^2^ test results showed no significant differences in sex composition or KL grading between the two groups. The *t* test results demonstrated no significant differences in age, BMI, or WOMAC scores. Table 2 shows that the Moses and *U*-test results indicate no significant difference in the dispersion range or mean value of the VL and VM elastic modulus values between the two groups when flexing and extending the knee joint at different angles. Table 3 shows that the *t* test indicates no significant difference in the daily average steps between the two groups in the first month, 2–3 months, and 4–6 months.

**Table 1:** Baseline of characteristics

	Gender(Female%)	BMI	KL grade(grade(*n*))	Age	WOMAC
EA + E	**80**	**23.56 ± 2.46**	**2 (18), 3 (12)**	**59.4 ± 10.30**	**74.67 ± 37.39**
E	**83**	**22.06 ± 2.46**	**2 (14), 3 (10)**	**59.92 ± 5.81**	**83.71 ± 32.52**
*p*	**0.825**	**0.791**	**0.930**	**0.126**	**0.626**

*EA* electrical acupuncture,* E* exercise

**Table 2:** Baseline of the elastic modulus values

	VM90	VM45	VM 180	VL90	VL45	VL180
EA + E-before	**8.87 ± 6.25**	**9.23 ± 3.88**	**10.62 ± 4.47**	**11.47 ± 5.51**	**14.13 ± 9.24**	**18.25 ± 10.43**
E-before	**9.15 ± 3.21**	**8.65 ± 4.69**	**10.70 ± 5.16**	**11.67 ± 3.85**	**10.77 ± 5.86**	**12.14 ± 5.14**
*p*	**0.134**	**0.288**	**0.134**	**0.134**	**0.134**	**0.134**

*VM* vastus medialis, *VL* vastus lateralis, *EA* electroacupuncture, *E* extensor training

**Table 3:** Daily average steps

	Steps (1 month)	Steps (2–3 months)	Steps (4–6 months)
EA + E-before	**2970.3 ± 452.84**	**2990.7 ± 259.36**	**2973.03 ± 252.45**
E-before	**3069.63 ± 351.03**	**2950.29 ± 237.66**	**2872.5 ± 241.94**
*p*	**0.230**	**0.746**	**0.997**

*EA* electroacupuncture, E* extensor training*

### Immediate and short-term effects of electroacupuncture combined with extensor training on the elasticity of quadriceps

**Fig. 2:**
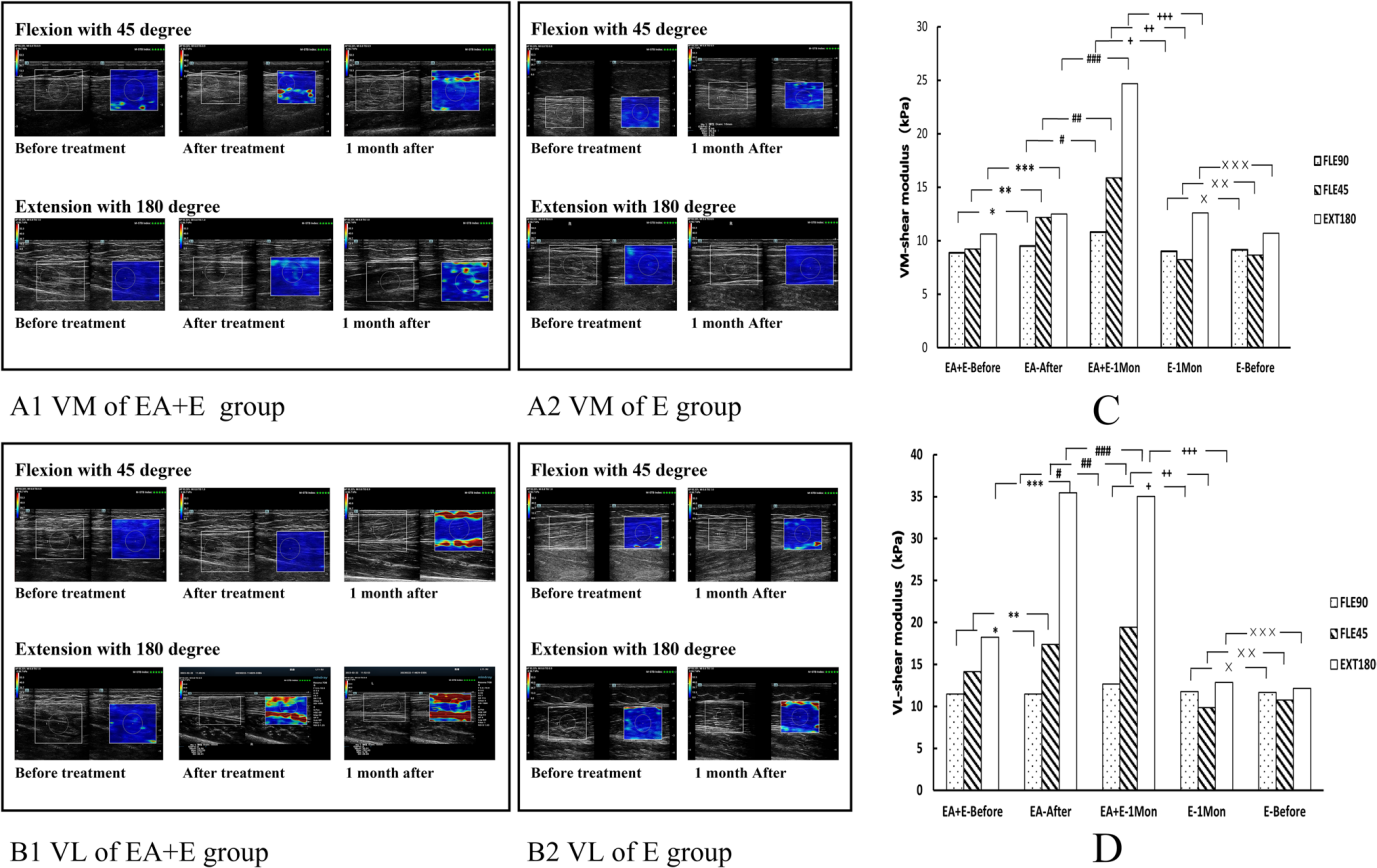
VM and VL elastic modulus values at different angles of knee joint flexion and extension at different time points. (**A1–2, C**): *p = 0.243, **p = 0.679,***p = 0.335; *#p* = 0.249, *##p* = 0.234, *###p* = 0.023; + *p* = 0.556, + + *p* = 0.009, + + + *p* = 0.002. × *p* = 0.902, × × *p* = 0.668, × × × *p* = 0.805. VL elastic modulus values at different angles of knee joint flexion and extension at different time points(**B1–2, D**): *p = 0.935,**p = 0.139, ***p = 0.001; *#p* = 0.802, *##p* = 0.745, *###p* = 0.647; + *p* = 0.715, + + *p* = 0.003, + + + *p* < 0.01; × *p* = 0.91, × × *p* = 0.813, × × × *p* = 0.861

The Moses test results showed no significant differences in the dispersion range of the elastic modulus values of the VM and VL before and immediately after EA. For the EA + E group, the *U*-test results showed a significant increase in the elastic modulus values of the VL with knee extension at 180° immediately after EA compared to before EA (Fig. 2, B1D). After 1 month, the elastic modulus of VM with knee extension 180° and the WOMAC scores were significantly higher than those immediately after EA (Fig. 2, A1, C). However, there was no significant change in VM and VL elastic modulus values and WOMAC scores in the E group after 1 month compared to the baseline (Fig. 2 A2 and B2, Fig. 4B). There was a significant difference in the elastic modulus values of the VM, VL, and WOMAC scores between the EA + E and E groups after 1 month (Fig. 2C, D). In summary, EA combined with extensor training can significantly improve the shrinkage elasticity value of the quadriceps and knee functions in a short period compared with extensor training alone.

### Long-term effects of electroacupuncture combined with extensor training on the elasticity of quadriceps

**Fig. 3:**
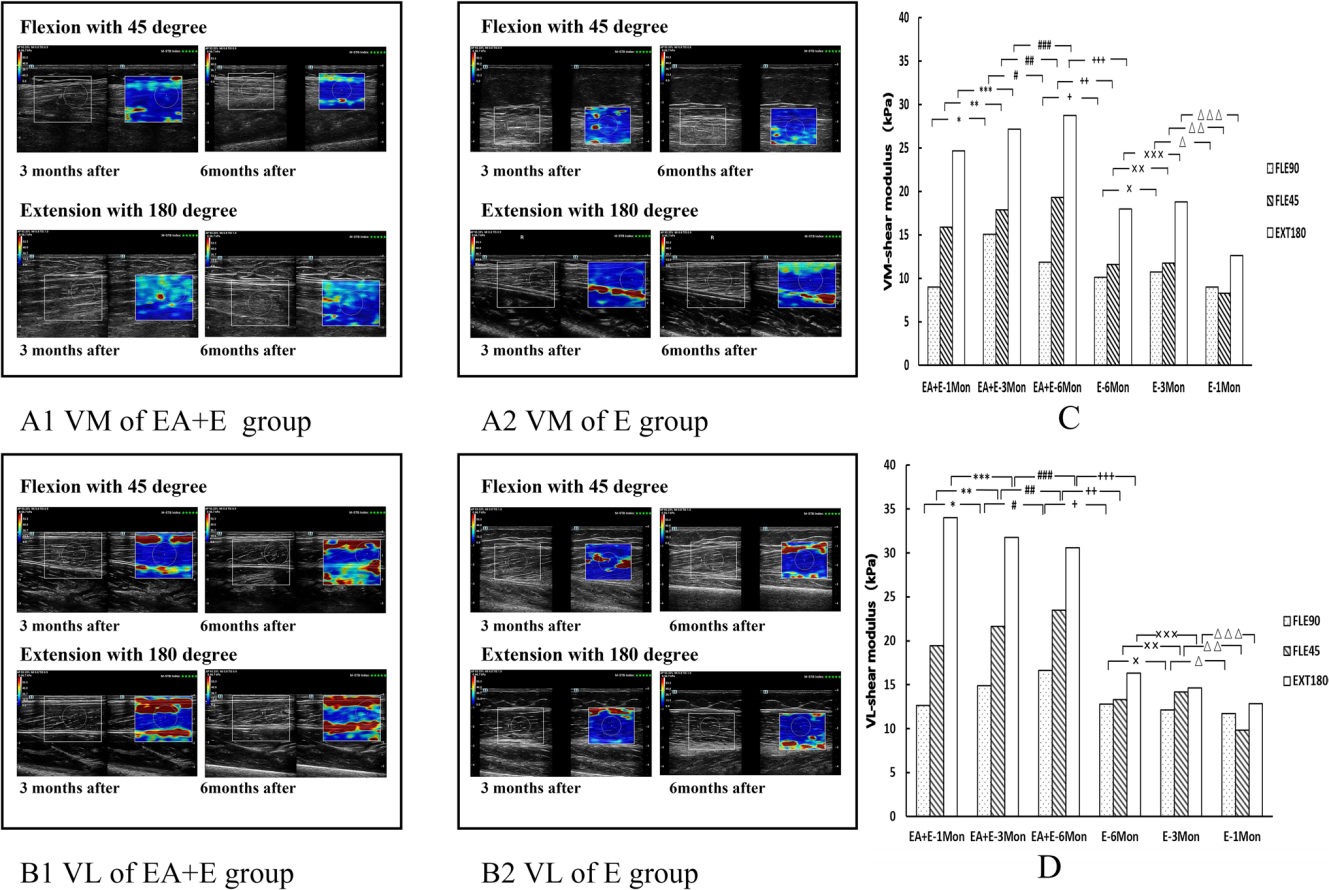
VM and VL elastic modulus values at different angles of knee joint flexion and extension at different time points. (**A1–2, C**): ^*^p = 0.345, ^**^p = 0.512,^***^p = 0.512; ^***#***^*p* = 0.838, ^***##***^*p* = 0.567, ^***###***^*p* = 0.806; ^**+**^*p* = *0.114,* ^**++**^*p* = *0.002,* ^**+++**^*p* = *0.041;* ^**×**^*p* = 0.932, ^**××**^*p* = 0.347, ^**×××**^*p* = 0.143; ^***△***^*p* = 0.843, ^***△△***^*p* = 0.008, ^***△△△***^*p* = 0.001. VL elastic modulus values at different angles of knee joint flexion and extension at different time points(**B1–2, D**): ^*^p = 0.595, ^**^p = 0.624, ^***^p = 0.567; ^***#***^*p* = 0.683, ^***##***^*p* = 0.486,^***###***^*p* = 1.0); ^**+**^*p* = *0.427,* ^**++**^*p* = *0.004,* ^**+++**^*p* = *0.01;* ^**×**^*p* = *0.755*, ^**××**^*p* = *0.41,* ^**×××**^*p* = *0.266*; ^***△***^*p* = 0.755, ^***△△***^*p* = 0.007, ^***△△△***^*p* = 0.128

**Fig. 4:**
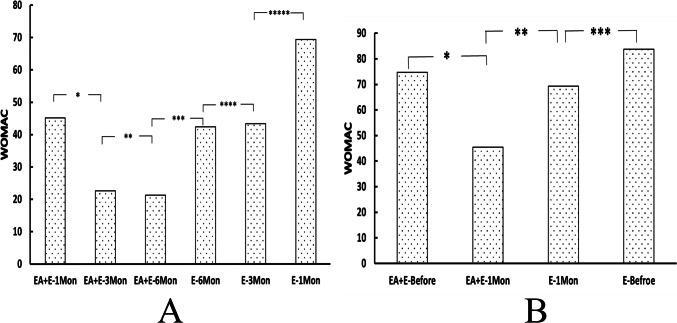
WOMAC rating at different time points: (**A**): *p = 0.03, **p = 0.87, ***p = 0.003, ****p = 0.918, *****p < *0.01;* (**B**): *p = 0.002, **p = 0.004, ***p = 0.101

The Moss test results showed no significant differences in the dispersion range of VM and VL elastic modulus values and WOMAC scores between the EA + E and E groups after 1, 3, and 6 months. However, the *U*-test results showed a significant increase in the elastic modulus values of the VM at 45° knee flexion and 180° knee extension in Group E compared to 1 month later. A significant increase in the elastic modulus values of the VL was also observed at 45° knee flexion (Fig. 3, A1–2, B1–2, C, D). The WOMAC scores of Group E decreased significantly after 3 months compared to 1 month ((Fig. 4A). After 6 months, the elastic modulus values of the VM and VL were significantly higher in the EA + E group than those in the E group, and the WOMAC scores were significantly lower in the EA + E group than those in the E group (Fig. 3, A1–2, B1–2, C, D, Fig. 4A). In group E, WOMAC score and the elasticity values of VL and VM after 3 months did not exhibit significant differences compared to those observed after 6 months (Fig. 3C, D, Fig. 4A). These findings indicate that EA combined with extensor training has better long-term efficacy in enhancing the contraction rigidity of the shrinkage elasticity value of the quadriceps and improving knee joint function than extensor training alone.

## Discussion

The results of this study confirm our hypothesis that the combination of EA and extensor training can significantly improve the contraction intensity of the quadriceps femoris in patients with OA in the short and long term compared with simple extensor training. In addition, EA combined with extensor training can enhance early functional recovery of OA. EA is a therapy that passes a microcurrent wave of human bioelectricity through a needle. A recent meta-analysis demonstrated that compared with other traditional acupuncture and moxibustion therapies, EA may be a better choice for OA regarding WOMAC pain and physical function scores [21]. Recent research has also shown that EA can activate specific peripheral and central nervous networks significantly reducing pain and improving stiffness and body function in patients with knee osteoarthritis [22]. Based on the results of this study, EA can significantly improve the contraction intensity of the VL muscle immediately and 1 month after EA. In contrast, the contraction intensity of the VM muscle only significantly increases 1 month after EA. This may be related to the different nerve and muscle contents in the VM and VL muscles of patients with OA. Fat infiltration in the VM is more severe than in the VL, which may explain why it cannot effectively improve the contraction strength of the VM immediately after EA. However, the combination of EA and training can significantly improve the contraction intensity of the VM muscle after 1 month. In contrast, simple extensor muscle exercise cannot significantly improve the contraction intensity of the quadriceps after 1 month, indicating the necessity of EA combined with extensor muscle training in the early treatment of OA.

In this study, the contraction intensity and WOMAC score of the quadriceps muscle in the affected knee of the control group exhibited a significant improvement at 3 months compared to 1 month. OA exercise therapy demonstrates a notable short-term effect. However, its maximum duration does not exceed 6 months, which aligns with findings from previous research [16]. This study found that continuing extensor training after EA can effectively maintain previous efficacy, and quadriceps muscle contraction function is significantly improved compared with simple extensor training. Based on prior research findings, microcurrent stimulation of human neuromuscles can promote the synthesis of local adenosine triphosphate, maintain intercellular calcium balance, and further optimize the structural and morphological changes in skeletal muscles caused by exercise. This can cause local hormonal changes, increase catecholamine secretion, and enhance exercise-induced lipolysis and skeletal muscle protein synthesis [23]. For example, microcurrent stimulation with current levels of 700–999 μA and a frequency of 10–25 Hz during 60 min of aerobic exercise can significantly increase fat breakdown in young men and women (18–30 years old) [24]. Another study revealed that microcurrent stimulation in patients with first-time anterior cruciate ligament rupture reduced skeletal muscle atrophy in the quadriceps femoris [25].In light of the considerations, it is speculated that EA combines microcurrents with acupuncture. Quadriceps femoris muscle acupuncture causes muscle fibers to wrap around the needle body, significantly enhancing the morphological adaptation and protein synthesis of local nerves and skeletal muscles during exercise. After EA treatment, continued extensor training can effectively maintain the protein content of the quadriceps femoris muscle and inhibit fat infiltration, thereby effectively consolidating the early therapeutic effect of EA, which is of great significance for the long-term stability of OA knee joint function.

The degree of knee joint pain is an important factor affecting OA joint function and the patient’s quality of life. Studies have shown that EA can alleviate mechanical hyperalgesia in both inflammatory and neuropathic pain models [26]. The PKCε-TRPV1(transient receptor potential vanilloid-1) signaling pathway in the dorsal root of the spinal cord is associated with pain transformation, and EA can inhibit the expression of PKCε and TRPV1 in the peripheral nervous system to prevent the transition from acute pain to chronic pain [27]. However, the activation of TRPV1 induced by skeletal muscle loading increases the intracellular calcium concentration and activates the mammalian target of rapamycin, promoting muscle hypertrophy [28]. In this study, EA combined with extensor training effectively improved WOMAC scores and ROM within 1 month, whereas simple extensor training took 3 months to improve significantly. The early application of EA is hypothesized to allow the passage of electrical signals through peripheral sensory nerves to the dorsal root of the spinal cord, leading to changes of PKCε and TRPV1 protein levels along the pain pathway. This modulation could potentially alleviate chronic pain caused by OA. Simultaneously, EA on the quadriceps femoris nerve muscle might cause the contraction load of the quadriceps femoris muscle, which may activate or upregulate the expression of TRPV1 in skeletal muscle, thereby alleviating the decline and atrophy of quadriceps femoris muscle function associated with OA. These changes may be consolidated by maintaining extensor training. In summary, further verification of the specific effects of EA and extensor training on the dorsal root of the spinal cord and quadriceps femoris is needed through animal and cellular experiments.

It should be noted that although EA is currently considered a safe and effective treatment method with minimal severe adverse reactions, the relationship between current intensity and frequency and muscle contraction intensity requires further validation through clinical and animal experiments. A study has shown that microcurrent stimulation with a current 1000 μa before exercise has no acute lipolysis effect [29]. Additionally, no research exists on the adverse effects and risks of using a safe range but a strong somatosensory current on the neuromuscular-skeletal muscle system. In this study, we applied a current of 0.8 ma to all patients. Some patients reported strong sensations and discomfort during the EA process, which were tolerable. No significant adverse reactions were observed after EA. Further clinical experiments are required for these patients to verify whether low-intensity currents can achieve the same effect.

Although the latest meta-analysis suggests that electroacupuncture is a potential effective method for treating OA, the current intensity and frequency used in various literature are not entirely consistent [18]. The selection of this study is mainly based on clinical practice. It is very important to standardize the selection range of current intensity and frequency. We will conduct a series of clinical randomized experiments in the future to verify the effect of different current and frequency choices on the contraction force of the quadriceps femoris muscle in OA. This study has certain limitations. While the body mass index (BMI) of the enrolled patients was below 28, it was not continuously monitored throughout the study duration. Changes in patients’ lifestyles may lead to an increase in BMI, which could further influence inflammation or symptom progression in osteoarthritis (OA), potentially introducing research bias.

## Conclusion

EA combined with extensor training can enhance the contraction strength of the quadriceps femoris and improve knee function in patients with short- and long-term OA. Therefore, the combination of EA and E has good application prospects and is recommended as a treatment combination for the better management of OA.

## Data Availability

The datasets used and/or analyzed during the current study are available from the corresponding author on reasonable request.

## Abbreviations

E: Extensor training
EA: Electroacupuncture
OA: Osteoarthritis
VL: Vastus lateralis
VM: Vastus medialis
